# The Overlap of Dissociative Identity Disorder and Narcissistic Features in an Adolescent: A Case Report

**DOI:** 10.1155/crps/1403303

**Published:** 2026-08-07

**Authors:** Buket Kılıç

**Affiliations:** ^1^ Department of Child and Adolescent Psychiatry, Şanlıurfa Balıklıgöl State Hospital, Şanlıurfa, Türkiye; ^2^ Department of Child and Adolescent Psychiatry, Hacettepe University, Ankara, Türkiye, hacettepe.edu.tr

**Keywords:** adolescence, case report, dissociative identity disorder, narcissism

## Abstract

Developmental trauma is a primary etiological factor in dissociative identity disorder (DID) and may lead to misdiagnosis due to symptom overlap with other psychiatric and neurological conditions. This case report describes an adolescent male with pronounced narcissistic vulnerability. The patient’s background features an inconsistent parenting style—characterized by both overprotection and severe criticism—and significant paternal pressure to achieve academic success and embody an idealized, wise persona. Chronic exposure to these psychosocial stressors is hypothesized to have contributed to acute narcissistic rage, potentially associated with dissociative identity fragmentation. The initial clinical presentation involved functional neurological symptoms, which progressed to distinct identity states with amnesia. Treatment focused on supporting the patient’s disrupted grandiose self‐structure through an empathetic, mirroring psychotherapeutic approach. This intervention was associated with a marked reduction in dissociative symptoms. Further research is needed to clarify the complex role of narcissistic vulnerabilities in the development of dissociative identity presentations.

## 1. Introduction

Dissociative identity disorder (DID) is characterized by the presence of two or more distinct personality states or identities, resulting in significant discontinuity in self‐perception, agency, and executive behavioral control. These changes are associated with concurrent variations in mood, behavior, consciousness, memory, perception, cognition, and sensorimotor functioning, observed by clinicians or reported by the individual. Individuals with DID also experience recurrent gaps in recalling everyday events, important personal information, or traumatic experiences that exceed ordinary forgetfulness [1]. The International Classification of Diseases, 11^th^ Revision (ICD‐11), similarly conceptualizes DID as part of the dissociative disorders spectrum, defining it by the presence of two or more distinct personality states with marked discontinuities in self and agency. In ICD‐11, these changes manifest as intrusive experiences or abrupt disruptions in identity, accompanied by recurrent episodes of dissociative amnesia [2].

The global prevalence of DID is estimated to approximately 1.5% in the general population [3, 4]. In clinical settings, this prevalence is higher; recent multicenter studies in psychiatric inpatient and outpatient units report dissociative identity presentations at rates ranging from 3% to nearly 10%, depending on diagnostic instruments [5]. Among adolescents, clinical data indicate an even higher vulnerability, with one study documenting a DID prevalence of 16.4% in a psychiatric outpatient clinic [6].

### 1.1. Etiology and Conceptual Frameworks of DID

The etiology of DID is primarily explained by two frameworks: the posttraumatic model and the sociocognitive model. The posttraumatic model, most widely accepted, posits that DID develops as a severe developmental defense in response to chronic early childhood abuse, neglect, or relational trauma [7, 8]. In this view, identity fragmentation protects the individual by isolating overwhelming traumatic memories from the primary self. In contrast, the sociocognitive model suggests that DID may arise from sociocultural influences, media exposure, and iatrogenic suggestibility in clinical contexts [9].

Although developmental trauma is central to dissociative pathology, examining concurrent personality structures and developmental pathways yields a more comprehensive perspective. The association between dissociative processes and narcissistic vulnerabilities has received increasing attention. Recent studies emphasize that early relational trauma and emotional neglect can promote both pathological dissociation and narcissistic defenses as adaptive responses to self‐fragmentation [10, 11]. Contemporary conceptualizations address the complex relationship between traumatic experiences and identity integration, highlighting systemic and sociocultural factors [12]. Modern psychotraumatology frameworks, such as dissoanalysis theory, clarify how environmental invalidation and denial trauma affect identity integration. This reveals a gap in the literature regarding nonclassic relational traumatizations manifesting as structural fragmentation during adolescence [13].

### 1.2. Treatment Paradigms of DID

Standard treatment guidelines for DID recommend a phase‐oriented approach. The initial phase emphasizes establishing safety, stabilization, and symptom reduction. The second phase focuses on processing and integrating traumatic memories, and the final phase aims for complete identity consolidation [14]. Common therapeutic modalities include psychodynamic psychotherapy, trauma‐focused cognitive–behavioral therapy, dialectical behavior therapy, and eye movement desensitization and reprocessing (EMDR). Pharmacotherapy is not a primary treatment for DID but is often used to manage co‐occurring psychiatric symptoms [15]. Recent clinical practice also supports integrative paradigms that combine trauma‐based alliance models to secure therapeutic attachment with self‐actualization approaches, thereby promoting identity consolidation beyond symptom management [12, 16].

DID is often misdiagnosed as borderline personality disorder, bipolar disorder, psychotic disorders, epilepsy, or somatic symptom disorders [17]. Self‐harming behaviors, suicide attempts, substance use, posttraumatic stress disorder, anxiety disorders, and major depression are frequently comorbid with DID [18]. Individuals with DID also commonly present with comorbid personality traits, including borderline, avoidant, obsessive–compulsive, narcissistic, dependent, and passive‐aggressive features [8, 19].

The relationship between DID and narcissism has been explored in a limited number of empirical studies. One study found a significant association between childhood physical maltreatment, dissociation, and grandiose narcissism, while vulnerable narcissism was linked to relational insecurity and depression [11]. Another conceptualization highlights that dissociation in narcissism has a strong relational component; in dissociative disorders, the breakdown of interpersonal and intrapsychic relationships, along with the structural dissociation of aggression and dependency, creates a rigid, closed system within pathological narcissism [20]. While research has addressed the intersection of dissociation, grandiose narcissism, and childhood maltreatment in adults [11], the interplay among these constructs during adolescence remains insufficiently studied. Adolescence is a critical period for identity consolidation, rendering narcissistic vulnerability and the grandiose self particularly susceptible to acute disruptions [21].

This case report presents a male adolescent with pronounced narcissistic vulnerabilities who was diagnosed with DID following a longitudinal psychiatric evaluation. Rather than replacing the prevailing trauma‐based etiology, this report proposes a complementary pathway: severe narcissistic injuries and an underlying fragile self‐structure may interact with relational stressors to trigger identity fragmentation in youth, even without explicit or retrievable histories of significant physical or sexual abuse. The aim is to demonstrate how developmental disruptions in the grandiose self can catalyze a dissociative identity split in an adolescent male and to underscore the clinical value of integrating a supportive, mirroring psychodynamic approach focused on self‐psychology and identity consolidation alongside standard developmental interventions for DID.

## 2. Case Presentation

### 2.1. Ethical Considerations and Consent

Written and verbal informed consent for the evaluation and publication of this clinical case were obtained from both the adolescent patient and his parents. To ensure confidentiality and protect the patient’s privacy, all personal identifying details were rigorously masked by omitting or altering nonessential biographical information. Formal institutional ethics committee approval was not required, as both Şanlıurfa Balıklıgöl State Hospital and Hacettepe University exempt individual retrospective case reports from formal review, and no experimental interventions were performed. Preparation and presentation of this study adhered strictly to the ethical principles outlined in the Declaration of Helsinki and followed the CARE guidelines.

### 2.2. Presenting Complaints

A 15‐year‐old male adolescent presented to the child and adolescent psychiatry outpatient clinic with his parents. The primary complaints included involuntary muscle contractions in the right arm, episodes of staring into space with a blank gaze, talking to himself, performing stereotypical or unusual movements, and periodic childlike regressive behaviors. Prior to this consultation, a comprehensive neurological evaluation—including neuroimaging and electroencephalography—was conducted. No organic etiology or epileptiform activity was found, prompting a referral to adolescent psychiatry.

### 2.3. History of Present Disorder

The involuntary muscle contractions in the right arm began 3 months prior to the admission. The clinical picture then progressed to episodic alterations in awareness. During these episodes, which ranged from 5–15 min to 2–3 h, the patient became unresponsive to his environment, gazed aimlessly, and exhibited automatic behaviors such as bringing his hand to his body, followed by coughing. He was observed talking to the wall as if deciphering a religious inscription, displaying severe irritability, or regressing behaviorally to the level of a 5‐ or 6‐year‐old child. During these states, he experienced identity confusion, asking, “Who are you?” and “Where am I?” These episodes were marked by complete localized amnesia: he retained no memory of his behavior, speech, or emotional state during these periods. Similar regressive and irritable episodes occurred at school, which were also obscured by amnesia. During the initial consultation, the patient was profoundly surprised when viewing family video recordings of these episodes, maintaining that he felt entirely healthy and recognized no stressors.

### 2.4. Family and Sociocultural Background

The patient lives with his parents and is the youngest of four siblings. His older sisters attend university, and his older brother works as a civil servant in another city. According to parental reports, the adolescent is generally cooperative and sensitive, with a strong tendency to internalize others’ problems. The birth history and developmental milestones were normal, and there is no history of chronic illness. The family noted that, as the youngest child, he was historically more attached to the home than his siblings. However, as he entered adolescence, he increasingly sought autonomy. His mother is a housewife with a chronic history of panic disorder managed pharmacologically, and his father works as a religious officer.

The family initially reported no significant domestic stressors affecting their child’s mental health. However, longitudinal interviews revealed a highly rigid and dysfunctional home environment, with parental approval being strictly conditional. The parents maintained uncompromising expectations regarding academic performance and sociobehavioral maturity, demanding that the adolescent always project a flawless, mature, and wise persona aligned with his father’s professional status.

His older sister reinforced this performance pressure by frequently shouting at him during academic tasks. In this environment, the patient developed a defensive style marked by chronic emotional internalization, suppressing anger to maintain a respectful stance. He described himself as highly emotional, tending to keep feelings inside and writing poetry and books in secret. When asked about emotionally significant events, he disclosed fondness for a girl whose picture he carried in his wallet, but he was unable to express his feelings because she was the daughter of his father’s close friend.

The patient’s academic history further contributed to his vulnerability. He attended primary school in a rural village for 3 years before transferring to a highly competitive city center, where he struggled with the curriculum and felt neglected by his new teachers. This led to a chronic pattern of academic failure. The persistent academic deficit conflicted with strict parental standards, causing the home environment to alternate between severe academic criticism and intense, intrusive parental overprotection.

Within this high‐pressure environment, two major external traumas occurred. The previous year, he was falsely accused of theft and defamed at school; under this ego‐destructive stress, he lost behavioral control and physically assaulted the accuser, severely damaging his relationships with teachers and peers. A few months prior to his admission, his paternal uncle died in a traffic accident—an event the adolescent deeply compartmentalized. He recalled meeting his uncle shortly before the accident, when the uncle asked them to stay longer, but they left early due to other commitments. Upon learning of the fatal crash, the patient became extremely upset and wept intensely during therapy sessions, though he interpreted the death as an unalterable matter of fate rather than experiencing personal guilt.

Currently, the adolescent harbors deep resentment toward his family’s historically condescending comments and has begun to respond with active frustration. To cope with profound feelings of inadequacy and academic failure, he maintains secret, grandiose ambitions related to history, politics, and military leadership, firmly believing that he is destined for significant achievements. He also reported recurrent dreams of snakes, which he interprets as hidden adversaries attempting to obstruct his path to success. He frequently fantasizes about achieving autonomy, similar to his older brother—living alone, working independently, and severing contact with his family—and occasionally imagines the deaths of his family members.

### 2.5. Diagnostic Process and Differential Diagnosis

During the initial psychiatric consultation, the mental status examination revealed a physically healthy adolescent male who appeared to be older than his stated age, demonstrating appropriate self‐care and meticulous grooming. The psychomotor activity was normal. The mood was euthymic, with a congruent and appropriate affect. Verbal associations were organized, goal‐directed, and linear. There was no evidence of formal thought disorder, loosening of associations, or active psychotic symptoms such as hallucinations or delusions. The immediate thought content was predominantly focused on school‐related subjects and academic stressors.

Despite an unremarkable baseline mental status examination, serial evaluations confirmed that the patient had not been functioning as his primary self for several months. Based on longitudinal assessment, he was diagnosed with DID according to DSM‐5 criteria. Clinical presentation met criterion A, with observable disruption of identity featuring distinct personality states (the primary self, a regressed 5‐year‐old child, and an irritable/aggressive alter) and fluctuations in affect and motor functioning. Criterion B was met by recurrent localized amnesia: the patient retained no memory of his behavior, speech, or emotional state during regressive episodes and outbursts of rage and demonstrated profound disorientation when viewing video recordings. Criterias C, D, and E were satisfied by marked clinical distress and academic impairment not attributable to substances, neurological conditions, or accepted socioreligious practices [3].

A systematic differential diagnosis was conducted to rule out overlapping conditions. Functional neurological symptom disorder was considered due to the initial arm contractions; however, structured identity alteration and amnesia supported DID as the primary diagnosis. Factitious disorder and malingering were excluded, as the patient showed genuine distress upon viewing video recordings of his attacks, his symptoms resolved during low‐stress individual sessions, and there were no apparent external incentives. Additionally, his strong desire to discontinue treatment to protect future eligibility for a military career contradicted any pattern of feigned illness or secondary gain [3].

### 2.6. Clinical Course, Psychotherapeutic Interventions, and Treatment

Psychiatric follow‐up was scheduled biweekly, but actual attendance stabilized at once a month. Initially, the patient exhibited significant secondary anxiety and shame regarding potential episodes at school. To address acute affective dysregulation, emergent anxiety, and severe behavioral outbursts during both conscious and amnesic states, pharmacotherapy was initiated with sertraline (50 mg/day) to stabilize anxiety and emotional distress and aripiprazole (5 mg/day) to augment impulse control and reduce dissociative rage. Concurrently, psychodynamically oriented supportive psychotherapy based on self‐psychology was implemented.

During the initial phase, as no spontaneous themes of sexual or physical abuse emerged, the therapist directly explored these domains; both the patient and his family explicitly denied any such trauma. A notable behavioral indicator during sessions was the patient’s consistent habit of carrying a briefcase‐like bag, reflecting an underlying need to project an idealized, respected adult persona. In response, the therapist adopted an empathetic, mirroring, and validating stance that supported the patient’s developmentally disrupted grandiose self‐structure, allowing him to feel respected in the therapeutic setting without resorting to dissociative defenses. As the therapeutic alliance deepened, the patient became able to express vulnerability more openly and spontaneously process themes of weakness, academic inadequacy, lack of respect, and anxiety regarding future high‐status roles.

A notable clinical contrast was observed during follow‐up: childlike regressive behaviors manifested consistently when the adolescent was accompanied by his family during joint sessions but were entirely absent during individual sessions with the psychiatrist. Parallel progress was observed in the family system after psychoeducation, suggesting that the family’s excessively protective and overbearing attitude actively reinforced the dissociative episodes. Collateral information from the patient’s sister revealed that, as the youngest child, he was historically spoiled, and while the father used strict language regarding academic underachievement, only the patient was severely affected. She also noted that when the parents were away from home and only the siblings remained, the patient experienced no dissociative episodes, confirming that the parents’ unintentional overbearing sensitivity reinforced the pathology. Additionally, his mother observed that the adolescent’s attacks primarily manifested as maladaptive coping mechanisms triggered by boredom, acute sadness, or a desire to elicit family affection.

Over the 1.5‐year follow‐up, conversion symptoms resolved completely, and dissociative symptoms were substantially reduced. Childlike regressive states became rare, significantly shorter, and occurred almost exclusively at home. Sporadic zoning‐out episodes at school were accommodated by peers, who regarded his condition as a neurological issue and shielded him from bullying. After a 6‐month symptom‐free period, aripiprazole was discontinued, and sertraline was stopped at 12 months. At the final follow‐up, the patient reported optimal well‐being and requested termination of treatment to protect legal eligibility for a future military career, with a mutual agreement to re‐engage if symptoms recurred. The timeline of the patient’s clinical course and treatment process is presented in Table 1.

**Table 1:** Concise timeline of the patient’s clinical course and treatment.

Timeframe	Clinical status and symptom progression	Interventions and outcomes
Premorbid period	Chronic emotional internalization and academic failure under rigid parental pressure	—
1 year prior	False accusation of theft and subsequent physical altercation at school	—
5–6 months prior	Tragic death of his paternal uncle	—
3 months prior	Onset of involuntary arm contractions	Neurological workup normal; referred to child psychiatry
Month 0 (admission)	Emergence of distinct identity states, behavioral regression, and localized amnesia	Sertraline (50 mg/day) + aripiprazole (5 mg/day) initiated; DID diagnosed
Month 1–5	Gradual reduction in symptoms; regressive states manifest only during joint family sessions	Mirroring/validating psychodynamic psychotherapy + family psychoeducation
Month 6	Resolution of functional conversion symptoms; stable 6‐month symptom‐free baseline	Ongoing psychodynamic psychotherapy; aripiprazole discontinued
Month 12	Sustained clinical and social stability; occasional school zoning‐out safely managed by peers	Sertraline discontinued; psychological identity consolidation maintained
Month 18	Complete structural stability and restoration of adaptive autonomous goals	Treatment mutually terminated at patient’s request to protect military career eligibility

Abbreviation: DID, dissociative identity disorder.

## 3. Discussion

The patient exhibited clear identity fragmentation and state‐dependent amnesia, meeting DSM‐5‐TR criteria for DID. The 2022 APA Text Revision notes that, while diagnostic thresholds remain stable, DID should be understood in relation to environmental and relational stressors [3]. In this case, symptoms began as a functional neurological symptom disorder with motor contractions, later progressing to dissociative identity episodes. Literature notes high comorbidity and overlap between conversion and dissociative disorders [22, 23], and affected individuals often struggle to verbalize their emotions and thoughts. Psychodynamically, conversion disorder represses affect into the somatic sphere (horizontal splitting), while dissociation enables contradictory ego states to coexist (vertical splitting), leading to compartmentalized awareness [24].

Regarding baseline personality traits, individuals diagnosed with DID often exhibit introversion, high internalization, and deep empathy [25], whereas conversion traits frequently involve high harm avoidance and lower self‐directedness [26]. In this case, the patient’s tendency to internalize reactions from a rigid upbringing, combined with feelings of academic inadequacy, appears to have predisposed him to both conversion and dissociation, with the clinical phenotype evolving from somatic conversion to structural identity fragmentation over several months.

There are two principal etiological models of DID: the trauma‐based model, which links DID to overwhelming childhood abuse or neglect, and the sociocognitive model, which sees DID as shaped by interpersonal influences and therapeutic suggestion [9, 27–29]. In this case, both the patient and family denied any history of physical or sexual abuse. While trauma‐related amnesia remains a possibility, it is also plausible that no severe abuse occurred. To reduce the risk of iatrogenic reinforcement, the clinician avoided naming alter states and described the condition as “a temporary disruption in identity integration related to a stressor.”

### 3.1. The Impact of Dysfunctional Family Dynamics

Given the absence of classic severe abuse, alternative psychodynamic and systemic pathways are considered, specifically focusing on narcissistic vulnerability. Rather than suggesting a direct causal link, this report adopts a hypothesis‐generating stance, proposing that profound narcissistic injury and chronic emotional invalidation may have significantly contributed to the clinical presentation and failure of identity integration. This formulation aligns with contemporary neurodevelopmental and psychodynamic models, which suggest that severe chronic environmental invalidation—unrelated to abuse—can destabilize ego boundaries in vulnerable adolescents [30, 31].

The patient’s home environment was characterized by rigid, conditional expectations from his father, a religious officer, and constant academic pressure from his sister. These dynamics led him to suppress his emotional needs and anger. After transitioning to an urban school and experiencing declining academic performance, he developed chronic feelings of inadequacy and “not being respected as a man.” In self‐psychology, chronic failures of developmental mirroring weaken self‐cohesion, increasing vulnerability to fragmentation [32, 33]. Systemic models also view such family dynamics as chronic emotional invalidation, reinforcing split‐off ego states to manage relational stress [13].

This fragile, unmirrored self‐structure was subsequently subjected to a series of acute external stressors that may be considered as alternative or compounding triggers for dissociative fragmentation. The sudden death of his paternal uncle in a traffic accident shortly after a brief family visit represented a major unintegrated loss. Although the patient denied conscious guilt and framed the death as fate, his intense weeping in the session indicates a profound, compartmentalized grief process. Literature suggests that individuals with pre‐existing structural vulnerabilities and unexpressed or complicated grief may experience acute traumatic triggers, accelerating dissociative splitting as a means to isolate overwhelming affective states [34, 35]. Furthermore, the false accusation of theft at school and subsequent loss of behavioral control (physically assaulting the accuser) inflicted an acute, devastating narcissistic rupture. This event shattered his internalized identity as a “respectful, flawless son,” alienated his peer group, and induced overwhelming shame. Shame, as opposed to guilt, involves a global condemnation of the self and is recognized as a potent catalyst for acute dissociative detachment in adolescents facing severe peer defamation [27, 36].

When severe environmental stressors—grief, academic failure, and social defamation—combined with persistent familial denigration, dissociative alter states likely emerged as defensive responses to manage these unresolvable conflicts. The childlike alter expressed regressive demands for unconditional paternal affection and safety, while the irritable state accommodated suppressed aggression provoked by parental criticism. This conceptualization aligns with the psychodynamic literature on the relationship between dissociation and narcissism. Brenner [25] described this interface through transitional objects, intersubjectivity, and the “false self,” emphasizing that structured identity dissociation can constitute severe character pathology at the extreme end of the dissociative spectrum. Similarly, Howell [20] argued that structural dissociation of aggression and dependency creates an internal, closed psychological system, disrupting adaptive interpersonal functioning. This perspective suggests that pathological narcissism can be understood as a relational and defensive form of dissociation. In this case, the patient, who had historically maintained a well‐adjusted, submissive persona and chronically suppressed outward anger to comply with parental expectations, developed dissociative identity fragmentation immediately after an explosive expression of narcissistic rage [37].

This progression shows that, in vulnerable adolescents, acute ruptures in a fragile self‐structure can lead to the emergence of distinct alter states. Howell [20] highlights the importance of secure attachments and empathetic interactions to support the safe expression of self‐protective aggression for structural recovery. Systemic vulnerability was corroborated by follow‐up observations: the patient’s regressive behaviors appeared only during joint sessions with his overprotective, anxious parents and were absent during individual sessions with the psychiatrist. Collateral information from his sister indicated that no dissociative episodes occurred when the parents were absent for several days. These patterns suggest an unconscious, systemically reinforced motivation linked to parental dynamics, where intense parental overprotection—partly driven by the mother’s chronic anxiety and panic disorder—unintentionally reinforced the patient’s regressive and conversion symptoms. This phenomenon is well documented in structural family therapy models addressing somatic and dissociative presentations [38, 39].

### 3.2. Therapeutic Interventions and Identity Integration

To address this structural deficiency, a significant portion of the psychotherapeutic intervention relied on an empathetic, validating, and mirroring stance derived from self‐psychology [32, 40]. By adopting a nonjudgmental tone, the therapist actively supported the patient’s disrupted grandiose self‐structure (as symbolized by his behavioral habit of bringing a briefcase to clinical sessions), allowing him to feel profoundly respected and helping him transform his secret, unrealistic ambitions into realistic, step‐by‐step goals. Regarding treatment outcomes, the robust reduction in the patient’s symptoms over the 1.5‐year follow‐up reflects a multifaceted synergy in which pharmacotherapy provided a supportive baseline for acute affective dysregulation, while the core therapeutic mechanism operated through a psychodynamic framework rooted in clinical self‐psychology and adolescent identity development [32]. By providing continuous empathetic mirroring and validation, the psychotherapist systematically addressed the patient’s underlying narcissistic fractures, facilitating the development of a more cohesive self‐structure [33]. This structural recovery reflects the core principles of modern self‐actualization models, in which the resolution of underlying narcissistic fractures enables the patient to transcend defensive fragmentation and attain autonomous personality integration [12]. Concurrently, this clinical scaffolding interacted synergistically with the natural developmental course of late adolescence, which inherently fosters ego identity integration [41, 42]. This integrated framework successfully allowed the patient to regain structural stability and pursue his adaptive, autonomous goals.

Although international consensus guidelines recommend a structured, phase‐oriented trauma framework as the standard approach for DID in adults [14], this case illustrates the potential advantages of clinical flexibility and developmental adaptation in adolescent populations. Several favorable prognostic factors enabled a more individualized intervention: the clinical profile was relatively mild, the fragmentation involved a limited number of alter states, and the patient demonstrated significant secondary anxiety and shame, reflecting preserved insight and ego‐dystonic friction. Additionally, as the patient was in middle adolescence, his ego identity formation retained substantial developmental plasticity. These factors suggest that when adolescent identity fragmentation is identified early and primarily results from structural validation deficits rather than severe chronic abuse, full recovery is highly achievable. In such early‐stage presentations, integrating systemic family psychoeducation with targeted developmental scaffolding provides a valuable and sufficient clinical pathway [43–45].

In conclusion, DID in adolescents remains a subject of ongoing etiological and therapeutic debate. A key limitation of this case is the reliance on clinical reporting, where trauma‐related amnesia might obscure a hidden history of early abuse. Due to clinical constraints, structured diagnostic interviews or standardized dissociation symptom scales were not used; thus, the diagnostic formulation relied primarily on longitudinal clinical observation and multi‐informant reports without independent verification. Nevertheless, this case suggests that a mild variant of dissociative identity fragmentation may emerge even in the objective absence of severe physical or sexual trauma; instead, it may be associated with chronic familial invalidation, severe narcissistic injuries, and unintegrated acute grief. Rather than minimizing the established role of developmental trauma, this case underscores the necessity of exploring more nuanced, nonclassic pathways to identity fragmentation. Further systematic prospective research is warranted to clarify the interplay between narcissistic vulnerability, systemic family dynamics, and dissociative states in adolescence.

## Funding

This study did not receive any financial support from any individuals or organizations.

## Conflicts of Interest

The author declares no conflicts of interest.

## Data Availability

Data sharing is not applicable to this article, as no datasets were generated or analyzed during the current study.
